# Eye of the Beholder: Stage Entrance Behavior and Facial Expression Affect Continuous Quality Ratings in Music Performance

**DOI:** 10.3389/fpsyg.2017.00513

**Published:** 2017-04-25

**Authors:** George Waddell, Aaron Williamon

**Affiliations:** Centre for Performance Science, Royal College of MusicLondon, UK

**Keywords:** performance, decision making, evaluation, multi-modal, continuous measurement

## Abstract

Judgments of music performance quality are commonly employed in music practice, education, and research. However, previous studies have demonstrated the limited reliability of such judgments, and there is now evidence that extraneous visual, social, and other “non-musical” features can unduly influence them. The present study employed continuous measurement techniques to examine how the process of forming a music quality judgment is affected by the manipulation of temporally specific visual cues. Video footage comprising an appropriate stage entrance and error-free performance served as the standard condition (Video 1). This footage was manipulated to provide four additional conditions, each identical save for a single variation: an inappropriate stage entrance (Video 2); the presence of an aural performance error midway through the piece (Video 3); the same error accompanied by a negative facial reaction by the performer (Video 4); the facial reaction with no corresponding aural error (Video 5). The participants were 53 musicians and 52 non-musicians (*N* = 105) who individually assessed the performance quality of one of the five randomly assigned videos via a digital continuous measurement interface and headphones. The results showed that participants viewing the “inappropriate” stage entrance made judgments significantly more quickly than those viewing the “appropriate” entrance, and while the poor entrance caused significantly lower initial scores among those with musical training, the effect did not persist long into the performance. The aural error caused an immediate drop in quality judgments that persisted to a lower final score only when accompanied by the frustrated facial expression from the pianist; the performance error alone caused a temporary drop only in the musicians' ratings, and the negative facial reaction alone caused no reaction regardless of participants' musical experience. These findings demonstrate the importance of visual information in forming evaluative and aesthetic judgments in musical contexts and highlight how visual cues dynamically influence those judgments over time.

## Introduction

The evaluation of performance quality is a fixture of musical practice. In educational and professional contexts, quality judgments are used to assess a performer's ability, to diagnose performance problems, to provide summaries of achievement, to determine competition rankings, and to award positions of employment (Goolsby, 1999). Reliable assessment tools are also vital to music research, used to determine the influence of factors such as self-efficacy (Ritchie and Williamon, 2011), practice quality and quantity (Williamon and Valentine, 2000), and the presence of an audience (Shoda and Adachi, 2014) on performance outcomes. These judgments are commonly provided by experts who are assumed to be able to provide consistent and objective indicators of quality. A growing body of research has examined this assumption, calling into question the reliability of expert judges' ratings (Wapnick et al., 1993, 2005), the consistency of interjudge agreement and rating severity (Wesolowski et al., 2015), the validity of the criteria used (Thompson and Williamon, 2003), raters' preexisting knowledge and impressions of the performer (Duerksen, 1972; Kroger and Margulis, 2017), and the influence of factors considered extraneous to traditional music performance quality judgment (for reviews, see McPherson and Schubert, 2004; Waddell and Williamon, 2017).

As live music performance often incorporates a visual element, many studies have explored the influence of visual cues on the perception of musical quality. Davidson (1993) showed that performers' body movements are not only indicative of their ability and expressive intentions but that participants are better able to differentiate such intentions when presented with the video alone, as opposed to those paired with audio or the audio alone. Further research has found visual performance cues to alter perception of violin vibrato (Gillespie, 1997), tone duration (Schutz and Lipscomb, 2007), and overall ratings of performance quality (Huang and Krumhansl, 2011; Lehmann and Kopiez, 2013; Morrison et al., 2014), including ratings of such predominantly aural concepts as phrasing, dynamics, and rubato (Juchniewicz, 2008). Quality evaluations have also been shown to be affected by otherwise unrelated visual features including race (e.g., Elliott, 1995; Davidson and Edgar, 2003; VanWeelden, 2004), dress (Griffiths, 2008, 2010, 2011), attractiveness (Wapnick et al., 1997, 1998, 2000; Ryan and Costa-Giomi, 2004; Ryan et al., 2006), and sex (Davidson and Edgar, 2003), while the introduction of the blind orchestral audition since the 1970s has been linked to a rebalancing of such biases, including a marked increase in the hiring of female performers (Goldin and Rouse, 2000).

These studies are supported by a meta-analysis that has demonstrated a global effect (*d* = 0.51 SDs) of visual information on performance quality, expressiveness, and appreciation ratings (Platz and Kopiez, 2012). Tsay (2013) provided a dramatic summation of this phenomenon. She gave participants 6-s clips of the three finalists in international piano competitions and asked them to identify the jury's top performer in each case. When provided with either audiovisual or audio-only information, the participants did no better than chance at selecting the winner, irrespective of musical training. However, those who were provided silent video clips identified the winner at a rate significantly higher than chance, a finding that was replicated with a second study using orchestral performances (Tsay, 2014). A key feature of Tsay's research was the use of very brief excerpts, forcing participants to form snap judgments of the recorded performances. The question remains as to whether the immediate influence of these visual features will persist over the course of an entire performance. This has been examined with the use of excerpts of varying lengths, although not with full performances and with conflicting findings. In supplementary studies, Tsay (2013) replicated her primary results using excerpts ranging from 1 to 60 s in length, suggesting that the effects may not be time-dependent. Research by Wapnick et al. (2009), however, found that the effects on ratings of some extra-musical visual attributes (attractiveness, dress, and stage behavior) varied as a function of excerpt duration (25, 55, and 115 s), although results were inconsistent between attributes and performers' sex. For example, high attractiveness significantly increased ratings for women only and only in the 25-s excerpts, while dress affected ratings for men only in the 25- and 115-s (but not the 55-s) excerpts.

One method of examining the long-term effect of visual information is by examining cues that are specific to one point in the performance, thus allowing for a residual effect to be studied after the cue is presented. The stage entrance provides such an opportunity, marking the time from when the performer emerges into the audience's field of view to the production of the first note, often incorporating a bow, acknowledgement of applause, and a brief preparation of the instrument (e.g., tuning, adjusting the seat). No music is being produced, thus any effect on evaluation of the subsequent musical material can be linked entirely to visual features. Platz and Kopiez (2013) compiled an inventory of 141 stage-entrance features drawn from previous studies, interviews with a small concert audience, and transcriptions of an acting tutor's commentary on select entrance videos. As stimuli, 27 videos of stage entrances were extracted from an international violin competition and manipulated to ensure consistent ambient audience noise (including applause) across conditions. Through appropriateness ratings of each video's entrance behavior on a 5-point scale by 435 participants across two preliminary studies, the corpus of 141 features was reduced to 56 and then to 10 salient behaviors via probabilistic test theory and item response theory models. In the final study, 1,002 participants rated the appropriateness of these 10 items while viewing 12 of the videos of entrance behavior and then indicated whether they would like to continue watching the ensuing performance. Of the 10 behaviors, six were found to be the most salient to judging the appropriateness of a stage entrance: nodding, direction of gaze, touching oneself, stance width, step size, and making a resolute impression. High-scoring entrances correlated positively with the viewer's motivation to continue watching. This suggests that the process of performance evaluation had already begun with the stage entrance and may have influenced perception of the musical content itself, although as the videos were stopped before the first note sounded, the effect on musical perception was not explicitly examined.

Musicians' facial reactions to specific performance events can also provide dramatic visual markers. The role of facial expression in music performance has been given greatest attention among singers, where studies have found their expressions to aid in lyric comprehension (Jesse and Massaro, 2010), to alter pitch perception (Thompson et al., 2005, 2010), to indicate musical phrasing (Ceaser et al., 2009) and to enhance emotional expression (Thompson et al., 2008; Quinto et al., 2014; Livingstone et al., 2015). However, facial expression has been experimentally examined far less in instrumentalists. Thompson et al. (2005) demonstrated that body and facial movements by blues guitarist B.B. King increased ratings of perceived aural dissonance by participants. In the context of the musical genre (the blues), this dissonance is expected, if not desired, thus the expression enhanced its effect. How then might facial expression influence the perception of inappropriate and unintended aural dissonance, such as when an explicit performance error has been made? Errors of pitch and timing are not considered trivial in the classical music tradition, although Repp (1996) found that only a relatively small percentage of errors in pianists' performances were noticed, even among highly trained listeners. This is to the performer's advantage; the goal should be to avoid drawing attention to a misplaced note or, if it has been detected, not to emphasize its importance. The role that facial expression plays in this process has not been systematically investigated.

In the present study, we tested the influence of visual cues on participants' quality ratings of music performances. We first examined stage entrance behavior, following the work of Platz and Kopiez (2013), to determine whether “appropriate” and “inappropriate” entrances indeed affect the perception of the musical content that immediately follows and whether such an effect lasts throughout the performance. We then examined the presence of facial reactions to a severe performance error. Performances were evaluated by representative samples of musicians and non-musicians, differentiated only by their level of musical experience. From these experimental manipulations and the existing literature, the following hypotheses were posited:

1A. The presence of an “inappropriate” stage entrance would cause a lower initial rating when compared with the same performance with an “appropriate” entrance. This first rating would also be made sooner, as a result of the performers' deviation from expected stage entrance behavior.

1B. As musically trained evaluators would have a stronger heuristic for “appropriate” stage entrances based on their extensive experience, they would show a shorter time to first decision and lower initial rating than the non-musician group.

2A. The addition of a severe performance error would cause an immediate decrease in performance ratings, measured from pre-determined points before and after the inserted error and the effect on the final rating when compared with a control performance. A corresponding negative facial reaction would intensify this response.

2B. As with hypothesis 1B, musicians' reactions to the error would be more severe than non-musicians' as a result of stronger expectations.

Testing these hypotheses required the measurement of participants' reactions to the performances as they unfolded, thus participants provided continuous responses in real-time using bespoke software in addition to completing overall, *post hoc* quality ratings. In order to maximize ecological validity, full performances were used that, despite experimental manipulations, gave the impression of live, undoctored performances.

## Materials and methods

### Participants

Participants (*N* = 105) with and without musical training were recruited via email and in person from conservatoires, universities, and public music and science festivals held in southeast England. Musicians (*n* = 53: 28 men, 25 women, mean age = 27.38, SD ± 12.16 years) were defined as participants currently undertaking undergraduate music training (*n* = 27), those completing or holding postgraduate music training (*n* = 23), and/or practicing professional musicians (*n* = 18). Participants not meeting these criteria were classified as non-musicians (*n* = 52: 31 men, 21 women, mean age = 30.82, SD ± 16.23 years), which included amateurs without specialist training (*n* = 30), participants who had undertaken some undergraduate training in music but did not currently practice (*n* = 6), and those who did not play an instrument or sing (*n* = 16), thus representing a variety of musical engagement. Primary instrument families represented across groups were piano (*n* = 30), string (*n* = 16), guitar (*n* = 11), woodwind (*n* = 11), voice (*n* = 7), brass (*n* = 6), and other (*n* = 6). The musician group had greater exposure to visually presented (live or recorded) classical performances, with 81% viewing at least monthly, in contrast to just 31% of non-musicians (13% of non-musicians reported never seeing performances). This study was conducted according to ethical guidelines of the British Psychological Society following internal Royal College of Music (RCM) approval on behalf of the Conservatoires UK Research Ethics Committee. Informed consent was obtained from all participants, and no payment was given in exchange for participation.

### Research design

**Figure 1:**
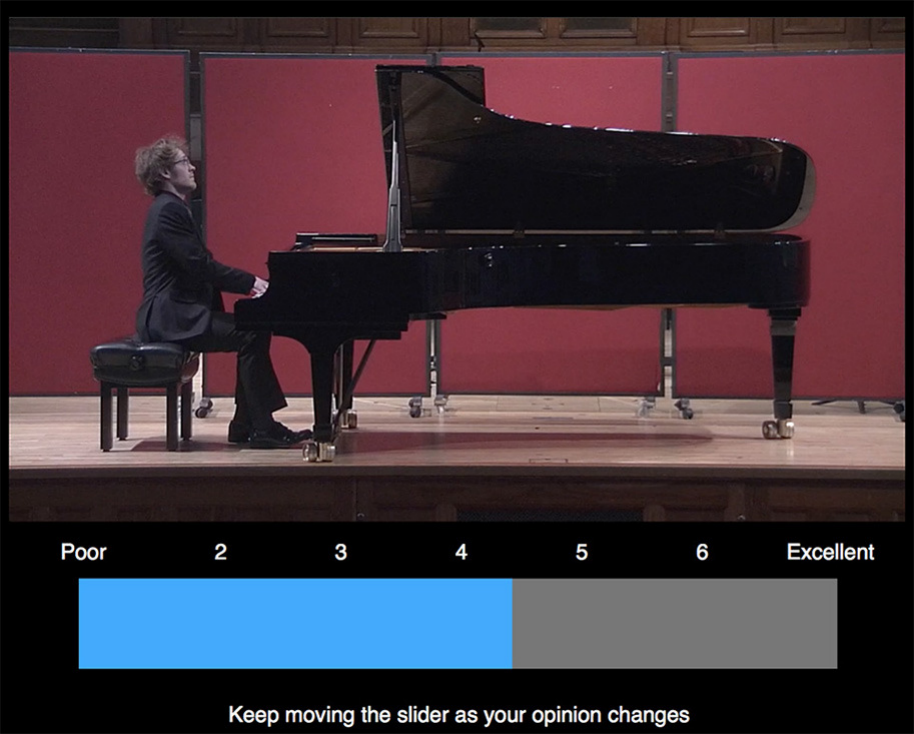
**Screenshot of the custom continuous measurement interface**. As the video plays the user can move the slider across the screen via the trackpad. Here, the user has already clicked to register the first judgment, turning the slider blue.

**Figure 2:**
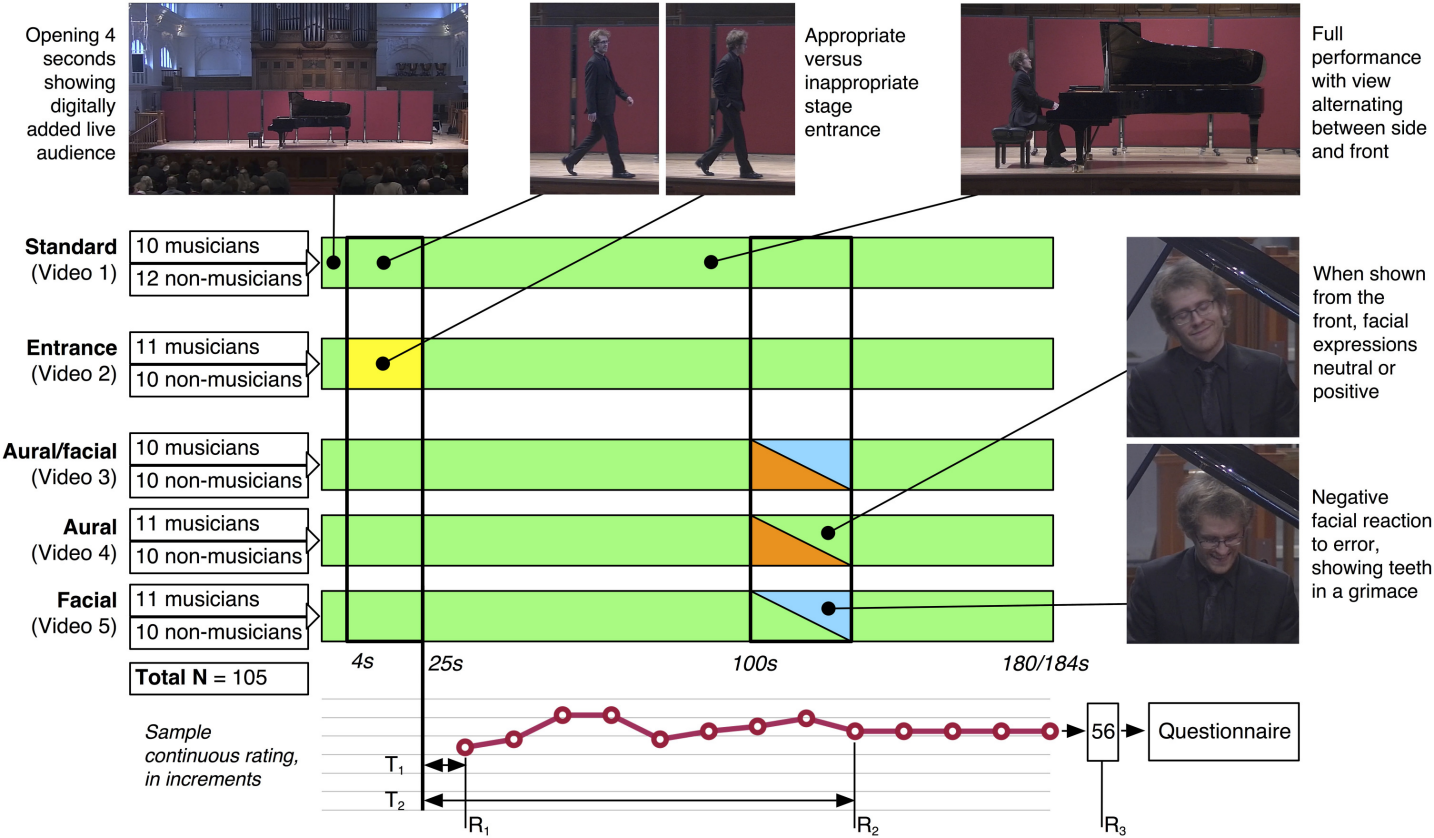
**The study design**. One hundred and five participants were each randomly assigned one video condition to view. From their continuous data, the time to first decision (T_1_), time to final rating (T_2_), first rating (R_1_), and final rating (R_2_) were calculated. The overall rating (R_3_) came from a written score completed after the video and continuous measurement were finished, followed by a questionnaire. Shading: yellow = inappropriate stage entrance, orange = aural performance error, blue = negative facial reaction.

Participants were randomly assigned to one of five conditions, each of which comprised viewing one of five videos of a manipulated piano performance while providing a continuous quality rating on custom software (see Figures 1 and 2). This was followed by a hardcopy questionnaire. Details of the stimuli, measures, and analyses are provided below.

### Stimuli

To maximize the ecological validly of the stimuli, recordings were created that would give the impression of a genuine live performance. Chopin's *Aeolian Harp* Etude (Op. 25, No. 1) was chosen as the work to be performed due to its short length (~3 min), its familiarity to Western classical audiences, and its homogenous structure: the composition features a perpetual-motion texture that is maintained throughout. Therefore, a brief break and resumption of that texture would be easily perceived by non-musicians as a severe unintentional error, similar in effect to a layperson with no knowledge of figure skating technique recognizing the severity of rare but occasional cases of professional skaters falling to the ice. A postgraduate pianist at the RCM performed the work in the RCM's Concert Hall on a grand piano. The lighting, staging, and performer's dress reflected a live concert experience. Audio was recorded via two Schoeps MK41 microphones hung above the stage, and video was recorded through two remotely controlled Panasonic AW-HE50 cameras.

Musicians have been shown to be highly sensitive to audiovisual asynchronies when viewing recordings of musicians with their hands in frame, particularly of their own instrument type (Bishop and Goebl, 2014). Therefore, footage of genuinely synchronized audiovisual information with the hands in view was cut with views wherein the hands were occluded during asynchronous moments. Camera 1 was positioned at the back of the hall and captured a lateral view showing the entire pianist and instrument including a clear view of the hands on the keyboard. Camera 2 was positioned at stage left, looking across the body of the piano with a clear frontal and tightly framed view of the performer's face and upper body, obscuring the hands. Behne and Wöllner (2011) demonstrated that such manipulations can give the impression of undoctored performances even among participants with high levels of musical training and knowledge of audiovisual and experimental manipulation techniques.

The pianist was instructed to perform the complete work from memory at a high, but not necessarily “perfect” standard, achieved by recording the work shortly before the performer considered it to be concert-ready. This resulted in several minor inconsistencies in the performance (e.g., a wrong note at ~128 s) maintained throughout each condition to increase the validly of such a performance containing a catastrophic error in the relevant conditions. Following the performance, the pianist bowed and walked off stage. The pianist was also recorded making two stage entrances: one appropriate and one inappropriate. These were based on the criteria outlined by Platz and Kopiez (2013), in which the appropriate entrance displayed a confident stride, repeated eye contact with the audience, a deep bow, and nods of appreciation for the applause, while the inappropriate entrance featured a narrow gate, limited eye contact, hands in pockets, and an abbreviated bow. Additionally, a performance error as described above was recorded in which the pianist was instructed to begin playing approximately two-thirds of the way into the piece (bar 27), and then make a critical error in which the performance stops for several seconds, he struggles momentarily to find his place, then continues onward. He was also given the explicit instruction to convey intense frustration at having committed the error through his facial expression. Finally, a wide shot was filmed displaying the set stage without the pianist present with the first several rows of audience seats visible. Previously recorded pre-concert activity in the same venue was then superimposed over the bottom section of the screen, along with corresponding audio, giving the impression of a live audience present for the performance. Audience applause (taken from existing footage from the venue to ensure acoustic validity) was added to the stage entrances and to the final bow. With the resulting footage, five conditions were constructed using Final Cut Pro 7, each exactly 3 min in length plus an additional 4 s in the two videos (3 and 4) containing an aural performance error (see Table 1 and Figure 2 for summaries and Videos 1–5 in the Supplementary Material).

**Table 1:** **Properties of the five videos used in the study**.

**Condition**	**Description**	**Stage entrance**	**Length (s)**	**Error at 100 s**
Video 1	Standard	Appropriate	180	None
Video 2	Inappropriate stage entrance	Inappropriate	180	None
Video 3	Aural error with facial reaction	Appropriate	184	Aural/facial
Video 4	Aural error only	Appropriate	184	Aural only
Video 5	Facial reaction only	Appropriate	180	Facial only

*Each video was formed of manipulations of the same recording of Chopin's Aeolian Harp Etude. Videos 1–5 are available in the Supplementary Material*.

### Continuous measurement

Continuous measurements, in which participants provide real-time feedback through a dial, slider, or software, have been used extensively in studies of music perception in relation to musical stimuli as they change over time, including listeners' genre preferences (Brittin and Sheldon, 1995), perception of loudness (Geringer, 1995), focus of attention (Madsen and Geringer, 1999; Williams et al., 2011), perception of musical intensity (Brittin and Duke, 1997), perceived tension (Madsen, 1998; Vines et al., 2006; Williams et al., 2011), perceived expressivity (Silveira and Diaz, 2014), and emotional responses (Madsen, 1998; Schubert, 1999, 2004; Nagel et al., 2007; Egermann et al., 2009). Such measures, however, have had relatively limited use in studies of performance evaluation. Himonides (2011) conducted a pilot study examining continuous quality ratings of sung vocal performances, and Thompson et al. (2007) established baseline values of time to first- and final-decisions and relationships between continuous and static ratings in judgments of short, audio-only piano performances. No research to date has examined whether such outcomes are affected by visual performance features as examined here. Thus, a bespoke tool was created using the software package *Presentation* (Neurobehavioral Software, v. 17.2) in order to deliver the video stimuli while simultaneously collecting synchronized continuous responses. After displaying an initial screen with instructions to “rate the quality of the following performance from ‘Poor’ to ‘Excellent,’ ” the software presented the video across the top of the screen. Underneath, a horizontal gray bar was presented alongside a rating scale ranging from 1 (poor) to 7 (excellent), following the scale used by Thompson et al. (2007). Horizontal movement on the laptop trackpad corresponded with a red bar moving across the gray space, which also recorded the position from 1 to 70 at 2 Hz in a separate file for analysis (also in line with Thompson et al., 2007). The red bar began at the midpoint (35 out of 70), and clicking the trackpad recorded a timestamp and turned the red bar to blue to confirm a first decision had been entered. Figure 1 displays a screenshot of the continuous measurement interface.

### Procedure

After providing informed consent, participants were told that they would be evaluating a recording of a classical pianist. They were instructed to base their ratings “not on how much you enjoy the performance, but by how ‘good’ you feel the performance is, as if you were a competition judge.” This differentiation was emphasized because the constructs of performance enjoyment and quality ratings, while correlated (Thompson, 2007), are assumed to be mutually exclusive in the act of professional performance evaluation (Thompson and Williamon, 2003). They were then able to try the continuous measurement software using a brief recording of a violinist playing unaccompanied Bach, with the instructions that:

- as soon as they had an opinion of the quality of the performance they should move the slider to the appropriate point and click (the click served to mark a first decision in the few cases where the slider's midpoint already indicated the participant's first rating), and
- they should feel free to move the slider at any point (without needing to click) if their opinion changed over the course of the performance.

They then initiated, watched, and rated one of the five videos (randomly assigned). Following the video, they completed a questionnaire on which they rated the performance's quality and typicality, their familiarity with the work, their enjoyment of the performance, and the appropriateness of the performer's on-stage behavior on 7-point Likert-type scales. They were also free to provide open comments on the performance. The questionnaire also collected basic background information including age and musical training.

### Data preparation and analyses

Data were first treated to several operations, primarily following Thompson et al. (2007), resulting in five general indicators of time to and score of first and final ratings. As a preliminary check, a visual examination of the data revealed one obvious erroneous spike in one participant's data caused by an accidental touch of the trackpad (i.e., a quick movement to an extreme score followed by an immediate return to the original score); this was removed and replaced with the score indicated immediately before and after the spike. Following this, five discreet variables were extracted from the full continuous data (see Figure 2):

- Time to first decision, **T**_1_: As a brief amount of time was necessary to move the slider to the desired first rating point, the time of first movement (or the first click in the 3 of 105 cases where there was no initial first movement) was noted as the initial decision time, T_1_. The continuous measurement ratings were taken from the beginning of the video, yet the first note was not played until 25 s; therefore, 25 s were subtracted from each score, giving initial ratings made prior to the first note a negative time value. Two outliers wherein a first decision was not registered until after two-thirds of the performance had elapsed were removed, based on an admission from one participant that she had forgotten to indicate any judgment until late into the trial.
- First rating, **R**_1_: The first point at which the participant maintained a stable rating of at least 2 s was taken as the first rating.
- Final rating, **R**_2_: The final score reported in the continuous data.
- Time to final rating, **T**_2_: Participants' continuous data tended toward brief, direct movements between stable plateaus. Thus, the time of final rating, T_2_, was recorded as the point at which the movement leading to the final rating (R_2_) was started. As with T_1_, 25 s were subtracted from each score to account for the stage entrance.
- Overall rating, **R**_3_: The overall written score provided in the questionnaire on a scale of 1–7. For a direct comparison with the final continuous rating, R_2_ was also converted from 70-point to 7-point values following Thompson et al. (2007).

Preliminary analyses using a series of *t*-tests showed no significant differences between men and women on the group T and R scores; subsequently, sex was discounted as a between-groups variable. Differences in R and T scores between conditions (Videos 1–5) and experience groups (musicians vs. non-musicians) were analyzed using 5 × 2 factorial ANOVA models. Planned contrasts were run specifically for the hypotheses being tested. In examining the effect of the stage entrance on T_1_ and R_1_ (i.e., hypothesis 1A), only Video 2 with the “inappropriate” entrance differed in opening material that could affect these measurements. Therefore, a Helmert contrast was employed as this allows a condition to be compared with the sum mean of the following conditions (i.e., Video 2 vs. 1, 3, 4, & 5; Video 1 vs. 3, 4, & 5; Video 3 vs. 4 & 5; Video 4 vs. 5). Simple contrasts, in which each video was compared with the *standard* control, were used for the remaining tests (i.e., hypothesis 2A). *T*-tests were used for direct comparisons of experience level in hypotheses 1B and 2B. As R_1_ and R_2_ were commensurable, they were tested using a mixed 2 × 5 × 2 ANOVA to examine changes between first and final ratings. To analyze moment-by-moment changes within each group resulting from the stage entrance behavior, performance errors, and facial reactions, repeated measures ANOVAs were calculated using mean scores at 10-s increments from the beginning of the video. This followed the method reported by Thompson et al. (2007), who used 15-s increments; the value was reduced to 10 to provide greater precision around the performance error.

## Results

Analyses in the first two sections below examine between-group differences (i.e., conditions 1–5 and musicians vs. non-musicians) and within-group comparisons of time to first decision (T_1_), time to final decision (T_2_), and first (R_1_), final (R_2_), and overall written (R_3_) ratings. A complete set of means and SDs are provided in Supplementary Table 1. The following two sections focus on repeated measures analyses of the continuous effects of the stage entrance and aural/facial errors. The final section examines relationships between general features of the participants' attitude toward the work, such as familiarity with and likeability of the piece.

### Effects of stage entrance on time to first decision (T_1_) and first rating (R_1_)

Four of the five conditions used the same opening material: that of the appropriate, confident stage entrance by the performer. Only the condition featuring the inappropriate stage entrance (Video 2) varied from the others in its opening material, thus we investigated whether participants responded differently to the altered stage entrance in both the time to and result of their first ratings: T_1_ and R_1_ (hypothesis 1A). To test this, ANOVAs comparing condition (×5) and musical experience (×2) with T_1_ and R_1_ as dependent variables were each followed by a Helmert contrast.

**Figure 3:**
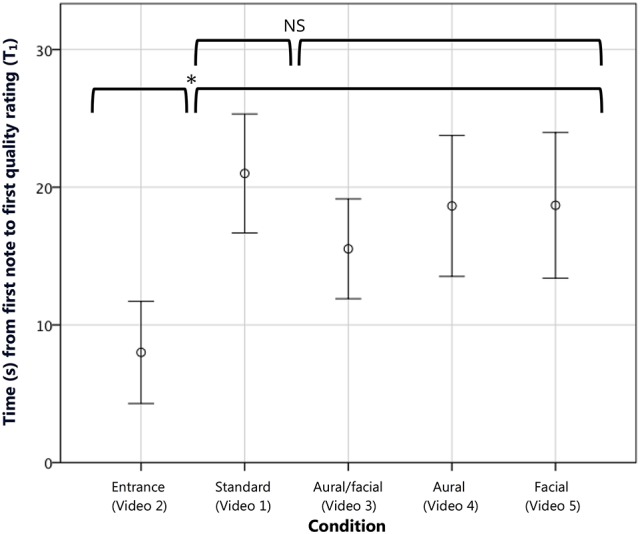
**The combined mean time to first judgment (T_1_) in seconds measured from the first note played**. The inappropriate *entrance* condition resulted in a significantly lower time to first decision compared with the other four conditions. Error bars show 95% CI. ^*^p < 0.05, as tested using a Helmert contrast in which the *entrance* condition was compared with the mean of all subsequent conditions.

For T_1_, while the ANOVA showed no overall differences between conditions, experience groups, or any interaction, the Helmert contrast showed a significantly lower time to first decision [*t*_(93)_ = −10.42, *p* < 0.05, *r* = 0.73] while watching the inappropriate stage entrance (*M* = 8.00, SD ± 17.00 s) vs. the combined effect of the remaining four (*M* = 18.52, SD ± 20.64 s; see Figure 3). Level 2 of the contrast, in which the *standard* condition was compared with the remaining three, showed no significant difference, demonstrating consistent decision times across groups viewing videos with identical opening material. Furthermore, 6 of the 21 *entrance* raters (29%) recorded a first decision before the performer had played his first note, compared with 6 of the remaining 84 participants (14%) that viewed one of the other four conditions.

**Figure 4:**
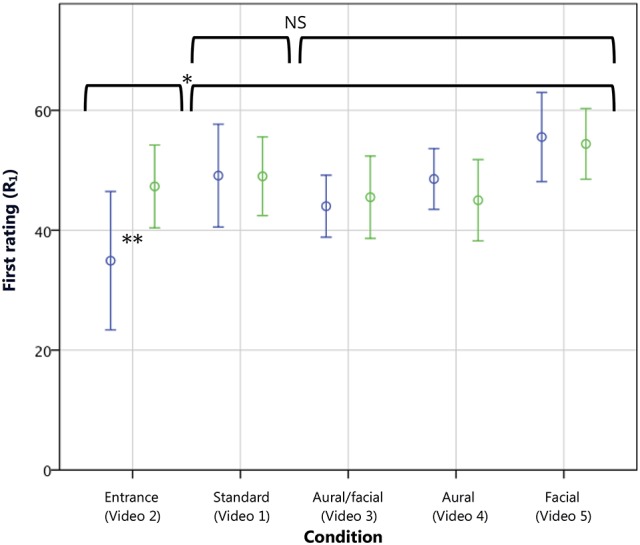
**First continuous ratings (R_1_) of musicians (blue) and non-musicians (green) on a scale from 1 to 70**. The inappropriate *entrance* condition resulted in a significantly lower first rating compared with the other four conditions. A direct comparison revealed that this difference was due to a significantly lower first rating among musicians as compared with non-musicians. Error bars show 95% CI. ^*^p < 0.005, as tested using a Helmert contrast in which the *entrance* condition was compared with the mean of all subsequent conditions. ^**^p < 0.05 in a comparison between musicians and non-musicians within the *entrance* condition.

For R_1_, the ANOVA showed a significant overall effect of condition [*F*_(4, 95)_ = 4.94, *p* < 0.005, η^2^ = 0.16], with no overall effect of or interaction with experience group. The Helmert contrast mirrored that of T_1_, showing a significantly lower score reported [*t*_(95)_ = −7.78, *p* < 0.005, *r* = 0.62; see Figure 4] by those watching the inappropriate stage entrance vs. the remaining conditions. Also as with T_1_, no significant effect was seen at the second contrast level comparing the *standard* and remaining videos. The hypothesis that musicians would more harshly penalize an inappropriate stage entrance (hypothesis 1B) was confirmed with a comparison [*t*_(19)_ = −2.00, *p* < 0.05, *r* = 0.42; one-tailed] wherein musicians gave an average initial rating of 34.91 (SD ± 17.18) and non-musicians a rating of 47.30 (SD ± 9.66), on par with first ratings across the other conditions. No significant difference in time to first decision (T_1_) was found between musicians and non-musicians in a similar comparison. Thus, the manipulated stage entrance was indeed found to have an effect on continuous quality evaluations. Musicians gave significantly lower initial ratings when viewing the inappropriate stage entrance, and both musicians and non-musicians delivered their first ratings of this condition in a significantly shorter length of time.

### Effects of condition on final decision (T_2_) and final rating (R_2_ and R_3_)

**Figure 5:**
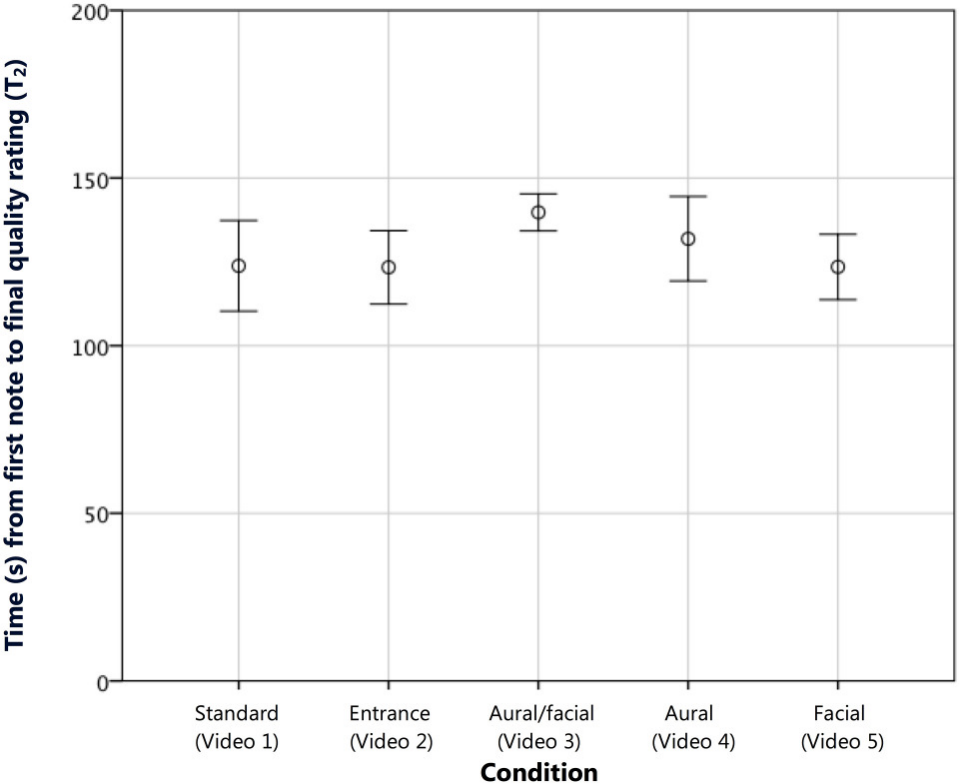
**The combined mean time to final judgment (T_2_) in seconds measured from the first note played**. No significant difference was found between conditions. Error bars show 95% CI.

The mean time to a final, stable rating (T_2_) across conditions was 128.31 s (SD ± 24.51) of the total 180 s of the entire performance (or 184 s for Videos 3 and 4, in which the aural error incorporated an extra 4 s of musical material). The ANOVA revealed no significant difference in final decision times based on condition or experience, and a Helmert contrast with the *entrance* condition in the first position and *standard* in the second showed no effect of condition at any level (see Figure 5). Thus, while the inappropriate stage entrance caused raters to make their first judgments more quickly (hypothesis 1A), it showed no significant effect on how long they took to come to a final decision about the performance.

**Figure 6:**
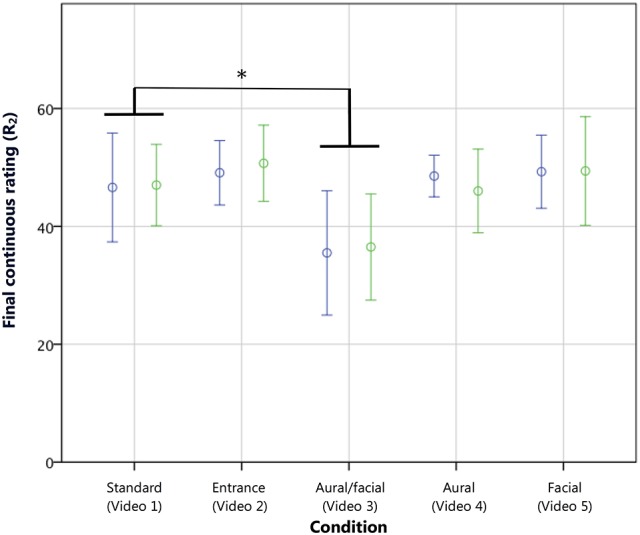
**Final continuous ratings (R_2_) of musicians (blue) and non-musicians (green) on a scale from 1 to 70**. The *aural/facial* condition, comprising a performance error with corresponding negative facial reaction, resulted in the only significantly lower performance rating. Error bars show 95% CI. ^*^p < 0.005, wherein a simple contrast compared each condition with the *standard*, with no interaction with experience group.

The mixed 2 × 5 × 2 ANOVA comparing the first (R_1_) and final (R_2_) continuous scores showed that, overall, the groups' initial mean ratings did not differ significantly from their final ratings. However, a significant interaction of rating and condition was shown [*F*_(4, 95)_ = 5.56, *p* < 0.001, η^2^ = 0.18], and a planned simple contrast comparing each condition with the *standard* showed that the *aural/facial* condition followed a different overall profile [*t*_(95)_ = −7.55, *p* < 0.01, *r* = 0.61]. As the ANOVA examining R_1_ showed no significant difference in the first score for this condition, it followed that a significantly lower final score would instead be the cause of the significant interaction effect. A 5 × 2 ANOVA examining R_2_ confirmed this with a significant effect of condition [*F*_(4, 95)_ = 5.56, *p* < 0.001, η^2^ = 0.19] with no effect of or interaction with experience. Again, a planned simple contrast was conducted comparing each condition with the *standard*. Only the *aural*/*facial* condition (*M* = 36.00, SD ± 13.37) was found to have received a final continuous rating significantly lower than the *standard* [*M* = 46.82, SD ± 11.55; *t*_(95)_ = −10.80, *p* < 0.005, *r* = 0.74; hypothesis 2A; see Figure 6].

**Figure 7:**
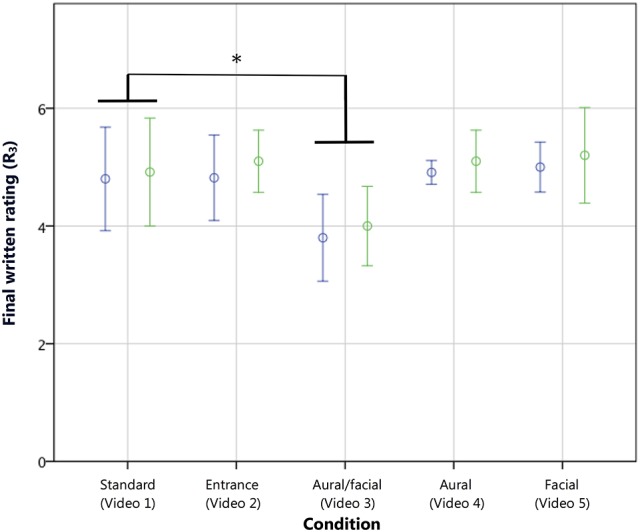
**Final written ratings (R_3_) of musicians (blue) and non-musicians (green) on a scale from 1 to 7**. As with R_2_, the *aural/facial* condition, comprising a performance error with corresponding negative facial reaction, resulted in the only significantly lower performance rating. Error bars show 95% CI. ^*^p < 0.005, wherein a simple contrast compared each condition with the *standard*, with no interaction with experience group.

An analysis of the final written scores (R_3_) showed similar findings, with a main effect of condition [*F*_(4, 95)_ = 4.87, *p* < 0.005, η^2^ = 0.17] and contrasts revealing that only the *aural/facial* score (*M* = 3.90; SD ± 0.97) was significantly lower than the *standard* on the 7-point scale [*M* = 4.86, SD ± 1.32; *t*_(95)_ = −0.96, *p* < 0.005, *r* = 0.10; see Figure 7]. A direct overall comparison of R_2_ and R_3_ with a repeated measures ANOVA (following a conversion of R_2_ from a 70-point to a comparable 7-point scale, as described in the “Data Preparation and Analyses” section) with experience and condition as between-subjects variables also showed no main effect of rating type on the reported scores. R_2_ and R_3_ also showed a strong correlation (*r*_τ_ = 0.70, *p* < 0.001). This suggests that the final continuous ratings accurately reflected the opinions given by the more routinely used written scores, thus confirming the validity of continuous rating as a proxy for evaluation scores given in standard summative procedures (Thompson et al., 2007). R_2_ and R_3_ both showed small correlations with R_1_ (*r*_τ_ = 0.23, *p* < 0.005 and *r*_τ_ = 0.23, *p* < 0.001, respectively).

These analyses found that the inappropriate stage entrance did not have a lasting effect on the final ratings (R_2_ and R_3_) given by either musicians or non-musicians. As this contrasts with the lower initial ratings (R_1_) given by musicians as reported in the previous section, the following section examines the point at which this difference in rating converged with the *standard* condition. Regarding the performance errors, only the *aural/facial* condition had a significant effect, lowering the final ratings (R_2_ and R_3_) of both experience groups. No overall effects of the *facial* or *aural* errors alone were found on the final ratings. Again, repeated measures analyses of the continuous measures data were then employed to examine the effect of the errors at the point of occurrence, as reported below.

### Continuous effects of the stage entrance

As the above analyses of the final and overall ratings (R_2_ and R_3_) showed that those viewing the inappropriate stage entrance condition did not yield significantly lower scores than those in the *standard* condition, the lower R_1_ scores reported by the musicians seem to have rebounded by the end of the performance. To identify how soon after the initial stage entrance this was accomplished, average ratings at 10-s intervals from the beginning of the video were extracted and analyzed using a repeated-measures ANOVA with planned contrasts of each interval to the final score. When conducted from the 50-s mark (25 s from the first note played), where 8 of the 10 musicians in this subsection were already reporting a mean score of 50.13 (SD ± 7.08), no significant difference from the final score was found in the remaining 12 levels. Thus, any negative impression caused by the inappropriate entrance, reflected in the quicker first rating among both experience groups and lower initial rating by musicians, was not reflected in the rating after 25 s of musical performance. Direct repeated-measures analyses prior to the 25-s point were not possible using this method due to the number of missing pairwise data sets resulting from participants who had not yet recorded their first rating. These results should be considered in light of the non-significant difference between the *entrance* and *standard* conditions in their change of R_1_ to R_2_, as shown by the 2 × 5 × 2 mixed ANOVA contrasts described above, where the difference in this subgroup did not emerge as significant when examined in conjunction with the other four conditions. Thus, any effect of the stage entrance on initial ratings among musicians did not persist when the pianist began playing, despite having formed their initial, more negative impressions significantly earlier.

### Continuous effects of the performance errors

**Figure 8:**
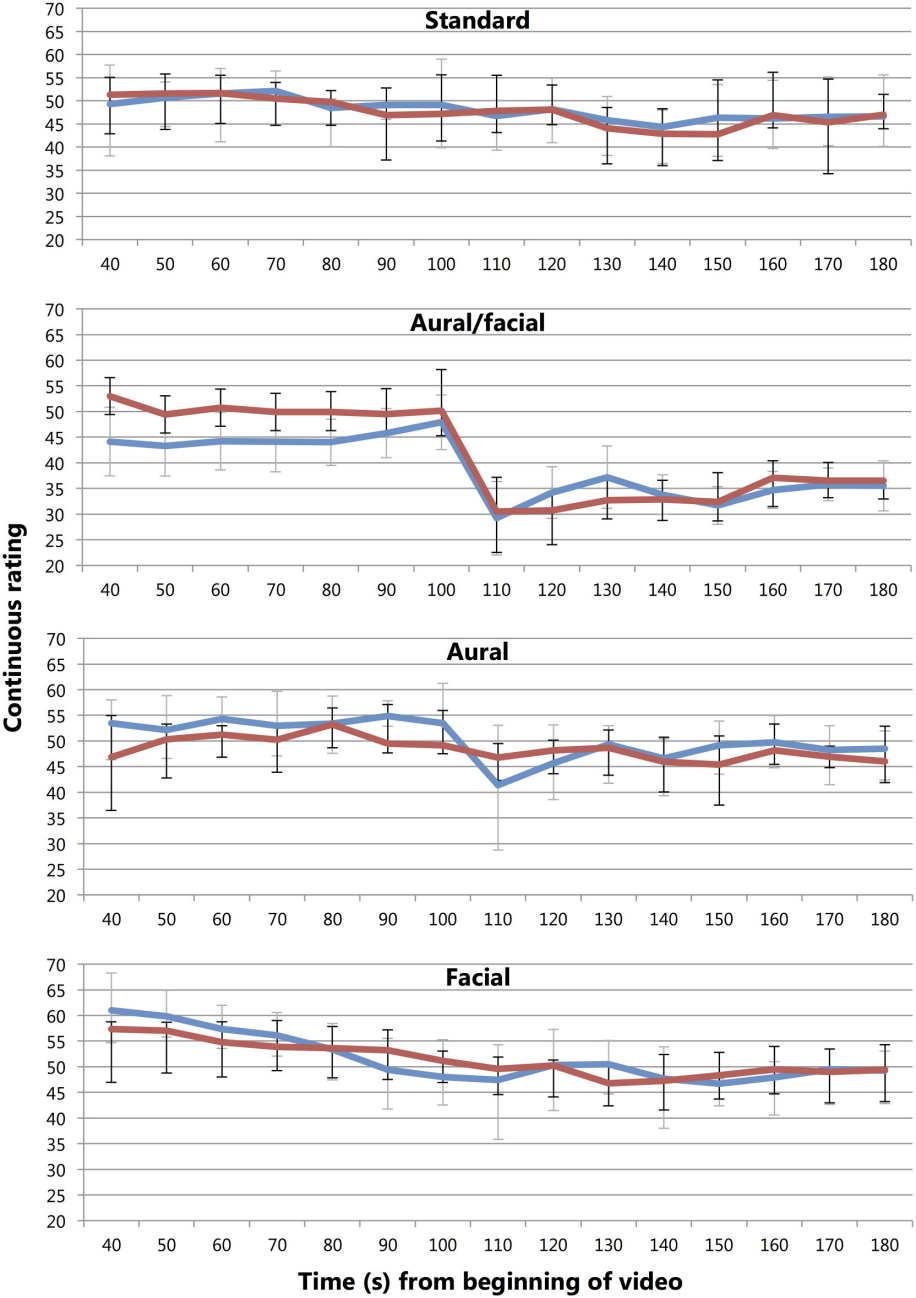
**Mean participant ratings of musicians (blue; gray error bars) and non-musicians (red; black error bars) across the *standard, aural/facial, aural*, and *facial* conditions at 10-s intervals**. Time in seconds from video opening—the first note was played at 25 s and the error occurred at 100 s. Axes begin at *t* = 40 s to reflect the point at which most participants were supplying data, allowing for consistent representation of mean and error. A larger drop can be seen at the point of the error in the *aural/facial* condition, with a smaller drop in the *aural* condition by musicians only and no significant movement in the *standard* and *facial* conditions. Error bars show 95% CI adjusted for repeated-measures data.

Three conditions related to performance errors: *aural/facial* (Video 3), in which a performance error with corresponding negative facial reaction was spliced into the *standard* recording (Video 1); *aural* (Video 4), in which audio from the same performance error was superimposed with the visual recording of the *standard* condition; and *facial* (Video 5), in which the visual reaction to the mistake was superimposed over the correct playing. As reported above, only the *aural/facial* condition triggered a significantly lower overall rating than the *standard*, reported by both musicians and non-musicians. Visual examination of the data revealed that this stemmed from a dramatic, immediate drop in continuous ratings immediately following the error by respondents when compared with the *standard* (see Figure 8).

To determine the individual and combined effects of the aural and visual (i.e., *facial*) components on musicians and non-musicians, average continuous ratings at 10-s intervals were again extracted and plotted. To determine when the final *aural/facial* score was finalized, a mixed ANOVA was conducted with 12 time intervals from the 70-s mark as a repeated-measures factor (30 s prior to the error, where 19 of the 20 participants across both experience groups had begun registering their continuous responses) and experience as a between-group variable. Planned contrasts comparing each point with the final score were used to isolate when the final decision was reached. A significant effect of rating over time was found [*F*_(11, 187)_ = 20.20, *p* < 0.001, η^2^ = 0.53] with no main effect of or interaction with experience, and contrasts were significant (*p* < 0.05, *r* = 0.32–0.62) until the 120-s point (20 s following the error) which followed a slight increase from the 110-s point following the error-invoked drop. To examine musicians' and non-musicians' specific reaction to the error, difference scores were calculated between ratings immediately before (100 s) and after (110 s) its presentation for the *standard, aural/facial, aural*, and *facial* conditions. The ANOVA revealed a significant effect of condition [*F*_(3, 80)_ = 14.85, *p* < 0.001, η^2^ = 0.28] with contrasts revealing that both the *aural/facial* condition [*t*_(80)_ = −19.02, *p* < 0.001, *r* = 0.90] and the *aural* condition [*t*_(80)_ = −7.25, *p* < 0.05, *r* = 0.63] showed significant drops in comparison with the *standard* (*M* = −19.20, SD ± 13.87; *M* = −7.43, SD ± 7.24; and *M* = −0.18, SD ± 8.07, respectively), but no such movement was seen in the *facial* condition (*M* = −0.90, SD ± 7.24; see Figure 8).

Hypothesis 2B posited that musicians would react to the performance error more severely than non-musicians. A comparison confirmed this in the *aural* condition where musicians made a significantly larger drop [*t*_(19)_ = −2.12, *p* < 0.05, *r* = 0.44] during that period, with musicians lowering their score by a mean 12.00 points (SD ± 12.69) and non-musicians by 2.40 points (SD ± 6.81) out of the total 70 over that 10-s period (see Figure 8). However, as shown by the R_2_ and R_3_ scores in the section above (see “Effects of Condition on Final Decision and Final Rating”), this penalization by musicians was not reflected in their overall ratings. No such difference was found in a similar comparison of the *aural/facial* condition.

To summarize, when the *aural* error was presented alone, the musicians reacted with a significantly lower immediate decrease in scores to the non-musicians, though this penalization was not reflected in the final scores. When the *facial* error was presented alone, no immediate or overall effect was shown, regardless of experience. When the two errors were juxtaposed in the *aural/facial* condition, however, both experience groups showed an immediate drop in continuous quality rating that was reflected in the final (R_2_ and R_3_) ratings.

### Work familiarity, likeability, and typicality

Participants' ratings of how much they liked and knew the composition (likeability and familiarity), how typical the performance was, and the appropriateness of the performer's behavior were tested for correlations (Kendal's tau, due to the large proportion of tied ranks within the 7-point scales) with T_1_, T_2_, R_1_, R_2_, and R_3_. After controlling for multiple comparisons, no significant relationships with the time to form their decisions (T_1_ or T_2_) were found, and only the appropriateness of the performer's behavior significantly correlated with the overall rating, R_3_ (*r*_τ_ = 0.28, *p* < 0.05), although its correlation with R_2_ was not significant and therefore should be interpreted with caution. A significant correlation between participants' familiarity with and liking of a composition was found (*r*_τ_ = 0.37, *p* < 0.01).

## Discussion

Music quality judgments, like the performances they seek to quantify, take place over time. The present study sought to examine this temporal nature of musical assessment, employing continuous measures methodologies to reveal previously unexamined immediate and overall effects on the decision-making process of extra-musical variables that could be defined by their having occurred prior to (i.e., the stage entrance) or at a specific point during (i.e., the error) a performance.

To achieve this, we manipulated a recorded performance of Chopin's *Aeolian Harp* etude to vary in appropriateness of the stage entrance or in the incidence of an aural performance error and/or corresponding negative facial reaction. We also examined the effect of experience by comparing response differences in musicians and non-musicians. The continuous ratings were able to show effects of these variations that the standard *post hoc* measurements would not have revealed. Where the inappropriate stage entrance did not have an overall effect on final ratings, the continuous data showed a significantly shorter time to first decision across experience groups and a lower initial rating by musicians that quickly recovered, confirming both hypotheses 1A and 1B. Regarding the errors, overall written scores showed that only the performance error with corresponding facial reaction (i.e., the *aural/facial* condition) led to a lower rating (hypothesis 2A), but the continuous measures data again demonstrated a more complex process at work. Musicians penalized then forgave a performance error on its own, only providing an overall score when the error was paired with a negative facial reaction. Non-musicians were significantly less harsh in their initial judgment of the aural error alone (hypothesis 2B), though behaved just as their more musically experienced counterparts when the facial reaction was juxtaposed. Neither group reacted to the negative facial reaction on its own.

In the discussion of their results, Thompson et al. (2007) questioned the generalizability of their finding that initial decisions were made within an average of 15 s following the first note, particularly in situations outside of their audio-only condition. The present research not only supports those findings, in that initial ratings across the four groups without the inappropriate stage entrance were made in approximately 18 s, but suggests that the presence of visual information relating to the performance, including the performer's behavior as they take the stage, does not alter this process to a great degree so long as the entrance is deemed “appropriate.” When stage entrances betrayed the expectations of their audience that decision was made earlier, and occasionally before the first note was played as was hypothesized by Thompson and colleagues. This study also found that experience did not play a role in the speed at which the judgment was formed, implying that the heightened expectations and knowledge of the material to be performed neither increased nor hindered the rate at which judges could form their decisions (or, at least, were willing or able consciously to record their first decision). However, experience did play a small role in the height of the first rating, where musicians reported a significantly lower initial score than non-musicians for the inappropriate entrance. Here, their greater experience with, and thus expectations of, the protocols of stage entrance behavior in the Western classical tradition may have caused them to penalize the performer more harshly. However, this judgment did not last long. When Platz and Kopiez (2013) demonstrated that the appropriateness of a violinist's entrance correlated positively with their anticipation of the performance's start, they wondered how sustainable the positive motivational effect might be were the performance to continue. While it is unclear what the specific effect of a *positive* impression might be in the current study, due to the finding that the average group ratings did not significantly differ from final ratings in the *standard* condition, it was shown that the *negative* impressions recorded by the musicians in the *entrance* scenario had dissipated (i.e., ratings had returned to the baseline of the *standard* rating) within 25 s of the first note. This aligns with the findings of Wapnick et al. (2009) where the visual effect of heightened attractiveness on higher quality ratings for female performers appeared in 25-s excerpts but not in longer ones. It is perhaps promising news for musicians; while the standard finding from the general evaluation literature is that negative first impressions are more resistant to change than positive impressions (e.g., Ybarra, 2001), in this case a negative first impression was quickly forgiven based on the quality of the performance that immediately followed. While stage entrance behavior made an impression on performance quality ratings, the impression of the musical content itself took precedence once it began. Future studies might examine the effect of an appropriate stage entrance on an initially poor musical performance, or whether a poor musical start can be as easily forgiven as the inappropriate stage entrance was here.

The negative impression of performance error on musicians was also temporary, with no indication in their final ratings that the error made a lasting impression. The lack of response from the non-musicians may indicate that they simply did not perceive that an error had occurred, though the severity of the mistake makes this situation unlikely. In the optional comments section, several non-musicians rating the aural condition indicated that they were aware of the error, where one wrote that she “perceived a mistake at about two-thirds of the way through.” Furthermore, the fact that non-musicians behaved in the same manner as the musicians in the *aural/facial* and *facial* conditions (i.e., reacting strongly to a performance error with negative facial response but having no reaction to the facial response on its own) indicates that they indeed perceived the aural difference. The question then remains why the facial reaction caused the error to be perceived as that much more detrimental to the performance, as when the negative expression was presented in isolation it caused no measured effect in either group. Put another way, it was not the behavior inherent to the expression that was penalized; it was how the expression altered the impression of the performance error itself. The ecological model of *emotion face overgeneralization* may account for this, wherein those interpreting a facial expression infer information not only concerning affective state but also of generalized traits (Zebrowitz and Montepare, 2008). Participants have rated people displaying sad faces as lower in trait dominance, while happy or surprised faces resulted in higher dominance and affiliation ratings (Montepare and Dobish, 2003). Thus, it could be expected that a musician's expression of frustration and anger at the committal of a performance error might result in the viewer regarding a trait tendency displaying general lack of control, instead of simply a performer who has, in that moment, lost control. Rather than being a musician momentarily making a mistake, they are perceived as musician *that makes mistakes*. This especially as the goal of music performance quality evaluations is often not only to rate the quality of the performance but, by extension, the performers themselves.

Both of these findings point to the interaction between aural and visual information, with the former taking some precedence. Tsay (2013, 2014) found that presenting visual information alone led to more accurate predictions of competition results than audio-only or audiovisual condition, though, crucially, participants were given extremely brief clips in which an immediate impression had to be formed. Here, a visually specific stage entrance caused an immediate reaction that was tempered after a period of aurally specific musical content, once participants were given time to process it. A visually specific facial reaction had no effect unless it supported an aurally presented musical error. While the visual element of performance still played a role, particularly in triggering immediate reactions, the aural information was dominant over time.

Generalizability of the present study is limited by the nature of the experimental condition. While the use of genuine performance recordings and video manipulation to give the impression of a live performance was undertaken to maximize ecological validity, participants nonetheless made their judgments in artificial situations, wearing headphones while observing the performances on a laptop screen. While many music quality judgments indeed take place in this environment, whether in private listening to a recording or professional evaluation of a recorded competition submission, whether the processes of evaluation here examined are maintained *in situ* during live performances, surrounded by fellow audience or panel members, remains unstudied. Furthermore, the use of multiple camera angles (necessary to hide the obvious asynchrony between the hands and music in the manipulations) maximized raters' view of the pianist's face at the point of the manipulated error in the relevant conditions. This provided ideal conditions for the effects of facial expression to manifest. While this framing is common in performance broadcasts, it is less likely to be viewable in single-camera or live performance settings and further study is required to determine whether the effects of facial expression are maintained in less ideal viewing conditions. It should also be noted that the presentation of inappropriate stage entrances or performance errors were inserted into a performance of particularly high (although not perfect) overall quality. This juxtaposition was intentional in order to provide a clear experimental framework, though further study will be required to determine whether an audience's tendency to “forgive” certain forms of performance error is maintained when the quality difference between those errors and the surrounding performance is not so stark. This also relates to the extreme severity of the performance error itself, where the performance momentary stopped. While common at amateur levels, this event is increasingly rare (but not unheard of) at such high ability levels. The current study demonstrates the effects of such a catastrophic mistake; further work could employ the same design with errors of varying nature and increasing subtlety. Finally, it could be argued that use of the continuous software interfered with participants' natural processes of performance evaluation, causing an increase in cognitive load that distracted from the final rating. Promisingly, when participants were asked following the experiment whether using the software consciously affected their ability to deliver a quality judgment, only 11% reported that it made the process more difficult; 46% reported that the software made no difference, and 42% reported that it made judgments easier. Schubert (2013) found a test-retest reliability of approximately 80% when using a continuous interface to record perceptions of musical emotion. A significant amount of unreliability stemmed from the opening of the performance, during which participants oriented themselves to the rating paradigm. The current methodology minimizes this issue in that participants were asked not to begin recording until they had decided on their first response. Overall, this suggests that familiarity with such devices in musical experiments does not significantly affect participants' ability to focus on the task.

Overall, the present study has demonstrated a temporally dynamic process of music performance quality evaluation that can be measured to determine the effects of temporally specific musical and extra-musical factors. Visual information in particular plays a key role in the decision-making process, but in a more nuanced relationship with the aurally based musical content than previous research has been able to demonstrate. In particular, the pre-performance rituals of Western classical performance made a difference on quality ratings, both in terms of impression formation and perhaps in determining performer traits. Whether or not it has been a focus of study, the role of personal expression on musical impression formation has been acknowledged for some time in practice. George Grove, the first director of the Royal College of Music and author of the eponymous Grove Dictionary of Music, was struck by such an effect when he saw the pianist Franz Liszt perform in 1886. He wrote that he “was delighted (1) by his playing, so calm, clear, correct, refined–so entirely unlike the style of the so-called ‘Liszt School’–(2) by his face. Directly he sat down he [sic] dismissed that very artificial smile, which he always wears, and his face assumed the most beautiful serene look with enormous power and repose in it. It was quite a wonderful sight” (Graves, 1903, p. 311). Grove was taken not only by the great pianist's performance, but the impression of Liszt's character; an impression that centered on the emotive capabilities of the face. Whether or not the visual aspect of Western classical performance has indeed been ignored in explicit practice and research, recent studies have moved it sharply into focus (e.g., Platz and Kopiez, 2012; Tsay, 2013, 2014; Silveira, 2014; Krahé et al., 2015). Continued study of these extra-musical variables and their effects on evaluation can now tease apart the relation between and weighting of their myriad aspects, the points in time at which each is most influential, and the lasting effects they may have as musical decision-making unfolds.

## Author contributions

All authors listed have made substantial, direct, and intellectual contribution to the work and approved it for publication.

### Conflict of interest statement

The authors declare that the research was conducted in the absence of any commercial or financial relationships that could be construed as a potential conflict of interest.
