# Supplementary material for: Eye of the Beholder: Stage Entrance Behavior and Facial Expression Affect Continuous Quality Ratings in Music Performance
Source: Front Psychol. 2017 Apr 25;8:513. doi: 10.3389/fpsyg.2017.00513 (PMC5403894; doi:10.3389/fpsyg.2017.00513)
Supplement: Supplementary file 1 [file Table1.DOCX]

Supplementary Material

Eye of the beholder: Stage entrance behavior and facial expression affect continuous quality ratings in music performance

**George Waddell and Aaron Williamon^*^**

Centre for Performance Science, Royal College of Music, London, United Kingdom

*** Correspondence:** Aaron Williamon, Centre for Performance Science, Royal College of Music, Prince Consort Road, London, SW7 2BS, United Kingdom. aaron.williamon@rcm.ac.uk

**Supplementary Table 1: Means, medians, and standard deviations** of T_1_ (time to first rating in seconds from first note), T_2_ (time to final rating in seconds from first note), R_1_ (first continuous rating score on 70pt scale), R_2_ (final continuous rating score on a 70pt scale), and R_3_ (overall score on a 7pt scale) for the musician and non-musician groups and 5 conditions (1 = *standard*, 2 = *entrance*, 3 = *aural/facial*, 4 = *aural*, and 5 = *facial*).

|  |  | *Overall (N=105)* | | *Musicians (n=53)* | | *Non-musicians (n=52)* | |
| --- | --- | --- | --- | --- | --- | --- | --- |
|  |  | *M (Median)* | *SD* | *M (Median)* | *SD* | *M (Median)* | *SD* |
| **1** | *T_1_* | 21.00 (14.75) | 20.26 | 24.25 (18.25) | 17.99 | 18.29 (14.75) | 22.39 |
|  | *T_2_* | 123.80 (134.25) | 30.44 | 114.00 (132.75) | 38.52 | 131.96 (140.00) | 19.87 |
|  | *R_1_* | 49.05 (48.00) | 10.84 | 49.10 (50.00) | 12.00 | 49.00 (46.50) | 10.33 |
|  | *R_2_* | 46.82 (47.00) | 11.55 | 46.60 (47.00) | 12.90 | 47.00 (46.50) | 10.88 |
|  | *R_3_* | 4.86 (5.00) | 1.32 | 4.80 (5.00) | 1.23 | 4.92 (5.00) | 1.44 |
|  |  |  |  |  |  |  |  |
| **2** | *T_1_* | 8.00 (7.00) | 17.00 | 7.50 (4.50) | 21.03 | 8.55 (7.25) | 12.26 |
|  | *T_2_* | 123.36 (127.00) | 24.00 | 126.86 (130.50) | 23.93 | 119.50 (124.25) | 24.74 |
|  | *R_1_* | 40.81 (43.00) | 15.16 | 34.91 (36.00) | 17.18 | 47.30 (46.50) | 9.66 |
|  | *R_2_* | 49.86 (46.00) | 8.40 | 49.09 (44.00) | 8.14 | 50.70 (51.00) | 9.04 |
|  | *R_3_* | 4.95 (5.00) | 0.92 | 4.82 (5.00) | 1.08 | 5.10 (5.00) | 0.74 |
|  |  |  |  |  |  |  |  |
| **3** | *T_1_* | 15.53 (13.00) | 16.22 | 13.90 (12.25) | 13.76 | 17.15 (17.50) | 18.98 |
|  | *T_2_* | 139.78 (140.0) | 11.79 | 140.25 (139.75) | 12.15 | 139.30 (141.25) | 12.06 |
|  | *R_1_* | 44.75 (42.50) | 8.31 | 44.00 (42.00) | 7.23 | 45.50 (45.50) | 9.61 |
|  | *R_2_* | 36.00 (36.50) | 13.37 | 35.50 (36.50) | 14.75 | 36.50 37.50) | 12.61 |
|  | *R_3_* | 3.90 (4.00) | 0.97 | 3.80 (4.00) | 1.03 | 4.00 (4.00) | 0.94 |
|  |  |  |  |  |  |  |  |
| **4** | *T_1_* | 18.64 (10.50) | 23.45 | 16.46 (10.50) | 25.72 | 21.05 (10.50) | 21.78 |
|  | *T_2_* | 131.88 (141.50) | 27.68 | 127.59 (137.00) | 25.76 | 136.60 (147.00) | 30.29 |
|  | *R_1_* | 46.86 (48.00) | 8.49 | 48.55 (48.00) | 7.54 | 45.00 (46.00) | 9.48 |
|  | *R_2_* | 47.33 (48.00) | 7.73 | 48.55 (48.00) | 5.26 | 46.00 (47.00) | 9.91 |
|  | *R_3_* | 5.00 (5.00) | 0.55 | 4.91 (5.00) | 0.30 | 5.10 (5.00) | 0.74 |
|  |  |  |  |  |  |  |  |
| **5** | *T_1_* | 18.68 (12.50) | 23.06 | 26.85 (13.50) | 29.76 | 9.61 (9.50) | 4.95 |
|  | *T_2_* | 123.48 (126.50) | 21.44 | 123.04 (126.50) | 24.39 | 123.95 (122.50) | 18.96 |
|  | *R_1_* | 55.00 (56.00) | 9.60 | 55.55 (52.00) | 11.07 | 54.40 (57.00) | 8.25 |
|  | *R_2_* | 49.33 (51.00) | 10.83 | 49.27 (51.00) | 9.22 | 49.40 (53.00) | 12.89 |
|  | *R_3_* | 5.10 (5.00) | 0.89 | 5.00 (5.00) | 0.63 | 5.20 (5.50) | 1.14 |
